# Metabolomic Profiling, Antibacterial, and Molluscicidal Properties of the Medicinal Plants *Calotropis procera* and *Atriplex halimus*: In Silico Molecular Docking Study

**DOI:** 10.3390/plants12030477

**Published:** 2023-01-19

**Authors:** Mostafa Y. Morad, Heba El-Sayed, Manal F. El-Khadragy, Asmaa Abdelsalam, Eman Zakaria Ahmed, Amina M. Ibrahim

**Affiliations:** 1 Zoology and Entomology Department, Faculty of Science, Helwan University, Helwan 11795, Egypt; 2 Botany and Microbiology Department, Faculty of Science, Helwan University, Helwan 11795, Egypt; 3 Department of Biology, College of Science, Princess Nourah bint Abdulrahman University, P.O. Box 84428, Riyadh 11671, Saudi Arabia; 4 Medical Malacology Department, Theodor Bilharz Research Institute, Giza 12411, Egypt

**Keywords:** medicinal plants, *Calotropis procera*, *Atriplex halimus*, GC/MS, antimicrobial activity, palmitic acid, schistosomiasis, *Biomphlaria alexandrina*, Wadi Degla Protectorate

## Abstract

The potential of plant-based natural compounds in the creation of new molluscicidal and antimicrobial medications has gained attention in recent years. The current study compared the metabolic profiles, antibacterial, and molluscicidal properties of the medicinal plants *Calotropis procera* (*C. procera*) and *Atriplex halimus* (*A. halimus*). In both plants, 118 metabolites were identified using gas chromatography-mass spectrometry. Palmitic acid, stigmasterol, and campesterol were the most prevalent constituents. *C. procera* extract showed stronger antibacterial activity than *A*. *halimus* against *Escherichia coli* and *Proteus mirabilis*. Both extracts exhibited molluscicidal activity against *Biomphalaria alexandrina,* with LC_50_ values of *C. procera* (135 mg/L) and *A. halimus* (223.8 mg/L). Survival rates of snails exposed to sub-lethal concentrations (LC_25_) of *C. procera* and *A. halimus* extracts were 5% and 20%, respectively. The hatchability of snail eggs exposed to both extracts has been dramatically reduced. Both extracts significantly decreased the levels of alkaline phosphatase, acid phosphatase, total protein, and albumin in snails, as well as causing DNA damage and resulting in numerous hermaphrodite and digestive gland damages and distortions. Molecular docking showed palmitic acid binding with acid, alkaline, and alanine aminotransferases in treated digestive gland snails. In conclusion, *C. procera* and *A. halimus* have antibacterial and molluscicidal properties.

## 1. Introduction

Schistosomiasis is a devastating infection that affects millions of humans and animals across the world [1]. According to a report by the World Health Organization published in January 2022, approximately 236.6 million people worldwide required treatment for schistosomiasis in 2019 (https://www.who.int/news-room/fact-sheets/detail/schistosomiasis (accessed on 10 November 2022)). *Schistosoma mansoni* is a parasitic trematode species that inhabits several African and South American countries and is regarded as the species that causes schistosomiasis. The intermediate host of this parasite is a freshwater snail, *Biomphalaria alexandrina* (phylum Mollusca, class Gastropoda) [2]. Snails have risen to prominence in both the medical and economic fields as a result of their participation in the spread of diseases that afflict a wide variety of animals [3]. Chemical methods of snail population control have several drawbacks, including high costs, toxicity to non-target organisms, and environmental accumulation [4], whereas biological snail population management can be inexpensive, safe, and more effective [5].

Because of the alarming spread of multidrug-resistant bacteria and the fact that microbiological infections are often fatal, the rapid development of new antibacterial metabolites is crucial [6]. The biological activities of natural chemicals originating from plants have led to the discovery and development of a substantial number of distinct medications; as a result, most of the current pharmacopeia’s effective medicines started as extracts from plants [7].

*Calotropis procera* is a member of the family Apocynaceae [7]. It is used traditionally to treat a variety of illnesses, such as diarrhea, leprosy, fever, and skin irritations such as eczema [8]. The plant’s leaf extract showed antidiabetic and antioxidant properties [9], and is used in treatments of rheumatoid arthritis disease [10]. Phytochemical constituents of plant leaves showed the presence of flavonoids such as soquercitrin, quercetin, and isorhamnetin [11].

*Atriplex halimus*, or saltbush, is a halophytic shrub of the family Amaranthaceae. This plant can survive in extreme environments, including salt, drought, and high temperatures [12]. Additionally, the plant is able to flourish in heavily metal-contaminated soil [13] and has been utilized in phytoremediation [14]. In folk medicine, the plant is utilized to treat cardiovascular disease, diabetes, and arthritis [12]. Chemical constituents of plants containing bioactive metabolites belong to various chemical classes such as flavonoids [15] and simple phenols [16].

*Calotropis procera* and *Atriplex halimus* have been used for traditional medicine for decades, and their capacity to thrive in challenging environments suggests that they may be rich in metabolites with yet-to-be-described activities.

In the current study, the chemical profiling, molluscicidal activity against *Biomphalaria alexandrina* snails, and antibacterial activities of the methanol extract of two medicinal shrubs, *Calotropis procera* and *Atriplex halimus*, were investigated.

## 2. Results

### 2.1. Metabolic Profiling of the C. procera and A. halimus Methanol Extracts

*C. procera* and *A. halimus* metabolic profiling was performed using a GC/MS apparatus. Fifty-two different metabolites have been successfully identified in *C. procera*; these metabolites include amino acids, sugars, sesquiterpenes, phenols, sesquiterpenoids, glucosides, saturated and unsaturated fatty acids, and sterols. Palmitic acid, campesterol, stigmasterol, oleic acid, and stearic acid were the predominant metabolites, accounting for 10.74, 8.48, 8.13, 8.04, and 4.24% of the plant extract, respectively. The amount of phytol compound was 3.91% of the total. In terms of sugars, sucrose (3.29%) and trehalose (3.23%) were the most abundant. L-proline was the most abundant amino acid in the extract, at 1.21%. Butanedioic acid and malic acid were the major carboxylic acids found in the extract. Regarding vitamins, α-tocopherol (vitamin E) and α-carotene (precursor of vitamin A) represent 1.34 and 1.76% of the total, respectively (Table 1). Both sesquiterpenoids and phenolic compounds made up 0.71 and 0.23 percent of the total.

Sixty-six compounds have been identified from the methanolic extract of *A. halimus*. Fatty acids, amino acids, sugars, and sugar alcohols are all represented among the metabolites. The fatty acids palmitic acid (6.47%), oleic acid (5.25%), and stearic acid (4.01%) constitute the majority of methanolic extracts. The second-most abundant component of the plant extract is sugars and sugar alcohols, including myo-inositol (5.14), glycerol (3.43), sucrose (2.24%), D-Fructofuranose (2.74%), and D-Pinitol (1.63%). The entire extract also contains a substantial number of organic acids, such as citric acid, which constitutes 4.05% of the total extract. Alanine was the most common amino acid among those identified, representing 1.18%. On the other hand, only trace amounts of sesquiterpenes have been found (Table 2).

### 2.2. Antibacterial Activity

Five distinct species of potentially pathogenic bacteria were used to investigate the antibacterial activity of the methanol extracts. Three different bacterial strains (*Escherichia coli* ATCC 25923, *Pseudomonas aeruginosa* ATCC 7853, and *Proteus mirabilis* ATCC 29906) were inhibited by an extract of *C. procera*, but only *Pseudomonas aeruginosa* ATCC 7853 was inhibited by an extract of *A. halimus* (Table 3). *C. procera* was more effective than gentamycin against *Pseudomonas aeruginosa* and *Proteus mirabilis*. However, neither extract inhibited the growth of *Staphylococcus aureus* or *Klebsiella pneumoniae*.

### 2.3. Molluscicidal Activity

The plant methanol extracts were tested for their efficacy against *B. alexandrina* snails, and both extracts demonstrated molluscicidal activity against the snails. According to the sublethal concentration LC_50_ (135 and 223.8 mg/L, respectively), *C. procera* extract exhibited higher activity than *A. halimus* extract (Table 4).

Over the course of four weeks, data on the survival rates of *B. alexandrina* snails exposed to sublethal concentrations (LC_25_) of *C. procera* methanolic extract (127.8 mg/L) or *A. halimus* extract (204.5 mg/L) were collected weekly (Table 5). Both extracts dramatically reduced snail survival when compared to the control group. After four weeks of exposure to *C. procera* and *A. halimus* extracts, the survival rate of snails was reduced to 5% and 20%, respectively.

*C. procera* extract was more toxic to snails than *A. halimus* extract. Similar results were recorded with the hatchability rates of eggs exposed to these sub-lethal doses of LC_25_ from plant extracts. The data showed that, compared to the control and *A. halimus* samples, the extract from *C. procera* considerably decreased the hatchability rate to 30% and increased the mortality of the snail’s eggs (Table 6).

**Figure 1:**
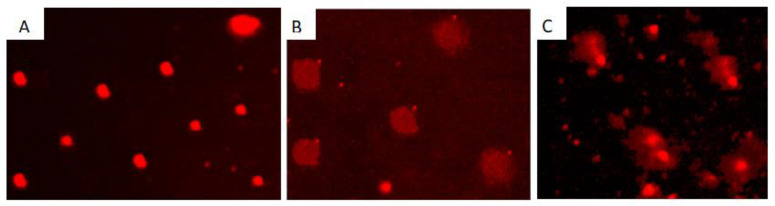
DNA single-cell damage in the digestive gland of *B. alexandrina* snails after exposure to sub-lethal concentrations of *Atriplex halimus* and *Calotropis procera* methanolic extractions. (**A**) Control (**B**) *Calotropis procera* (**C**) *Atriplex halimus*.

The exposure of *B. alexandrina* snails to sub-lethal concentrations of *C. procera* or *A. halimus* methanolic extracts caused obvious DNA breaks, as revealed by the percentage of the comet, tail length, percent DNA in tail, and tail moment, which were increased (*p* < 0.05 and 0.01) compared to control snails (Figure 1 and Table 7).

Sub-lethal doses (LC_25_) of methanol extracts of the studied plants had a biochemical effect against *B. alexandrina* snails. Alkaline phosphatase concentration was decreased to 75.4 ± 0.1 and 60.5 ± 0.3 μmoles/mg following exposure to *A. halimus* and *C. procera* extracts, respectively. Comparatively, acid phosphatase concentrations were dramatically decreased following exposure to LC_25_ plant extracts compared to the control group. In addition, the concentrations of total protein and albumin have reduced, and the level of alanine aminotransferase has increased dramatically to 88.5 ± 0.6 and 107.2 ± 0.4 U/L, with sub-lethal quantities of *A. halimus* and *C. procera*, respectively (Table 8).

**Figure 2:**
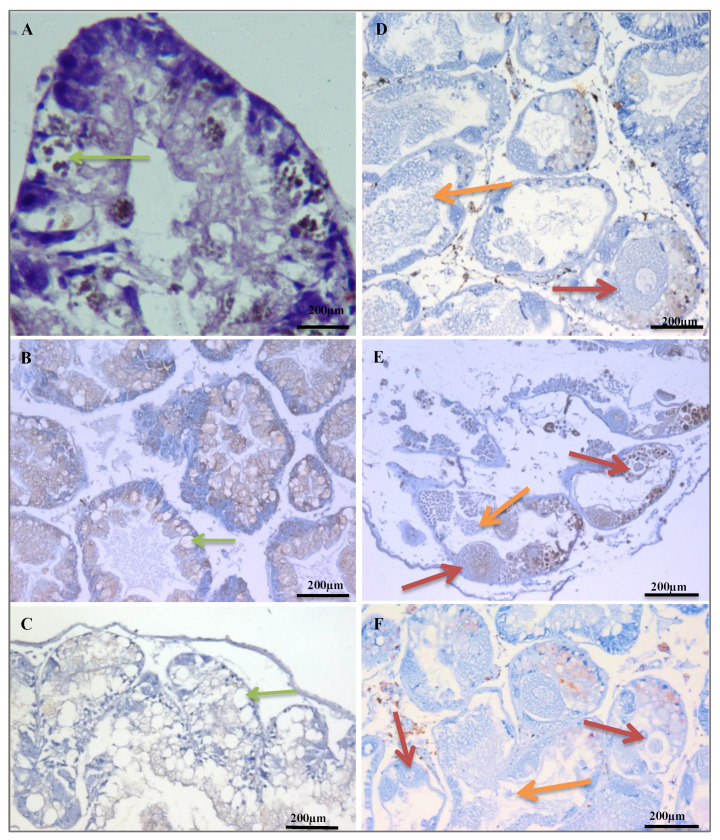
Light micrographs of the digestive (left side) and hermaphrodite (right side) glands of *B. alexanderina* snails. (**A**) Digestive gland of control *B. alexanderina* snails showing no expression of cyclin D1, the digestive cells found in a digestive follicle showing normal structure (green arrow) (H&E, 400). (**B**) Digestive gland of exposed snails to LC25 (127.8 mg/L) of *C. procera* methanolic extraction showing expression of cyclin D1 (brown stained parts) (Immunohistochemistry for cyclin D1, ×100). Degeneration of some digestive cells and appearance of vacuolations (green arrow). (H&E; ×100). (**C**) Digestive gland of exposed snails to *A. triplex* showing low expression of cyclin D1 (Immunohistochemistry for cyclin D1, ×200). Degeneration in digestive cells showing many vacuolations (green arrow). (**D**) Hermaphrodite gland of control *B. alexanderina* snails showing no expression of cyclin D1, mature ovum found in a female follicle (red arrow), spermatozoa found in the center of a male follicle (orange arrow), (H&E; ×200), (Immunohistochemistry for Cyclin D1, ×200). (**E**) Hermaphrodite gland of exposed snails to LC25 (127.8 mg/L) of *C. procera* methanolic extraction showing expression of cyclin D1 (brown stained parts) (Immunohistochemistry for cyclin D1, ×200). Degeneration of mature ovum (red arrow), and sperms in the center of a male follicle (orange arrow) (H&E; ×200). (**F**) Hermaphrodite gland of exposed snails to *A. triplex* showing mild expression of cyclin D1 (brown stained parts) (Immunohistochemistry for cyclin D1, ×200). Degenerated mature ovum (red arrow) and sperms (orange arrow) (H&E; ×200).

Inspecting the histological sections of *B. Alexandrina* demonstrated that the digestive and hermaphrodite characteristics of the control sample were distinct from those of the experimental sample. The digestive gland of control snails displayed normally distributed digestive tubules with digestive cells composing each follicle (Figure 2A). Degeneration and vacuolation of many digestive cells as well as high expression of cyclin D1 were observed in snails that were exposed to a methanolic extract of *C. procera* (Figure 2B). Whereas the hermaphrodite gland revealed the presence of a male acinus with numerous spermatozoa in the center and a female acinus with mature ovum in the follicle center (Figure 2D). Additionally, the hermaphrodite gland was severely damaged, and both the male and female acini represented cyclin D1 on spermatozoa and mature ova (Figure 2E). Whereas cyclin D1 expression was undetectable in both glands in the control groups, 70% and 40% expression were found in the interstitial cells after exposure to *C. procera* and *A. halimus* extracts, respectively. Compared to snails exposed to *C. procera*, those treated with a methanolic extract of *A. halimus* had less impact on the digestive and hermaphrodite glands. However, in the case of exposure to *A. halimus* degeneration of digestive cells, the presence of vacuolated types and low expression of cyclin D1 in the digestive and hermaphrodite follicles were observed (Figure 2C,F).

### 2.4. Molecular Docking

**Figure 3:**
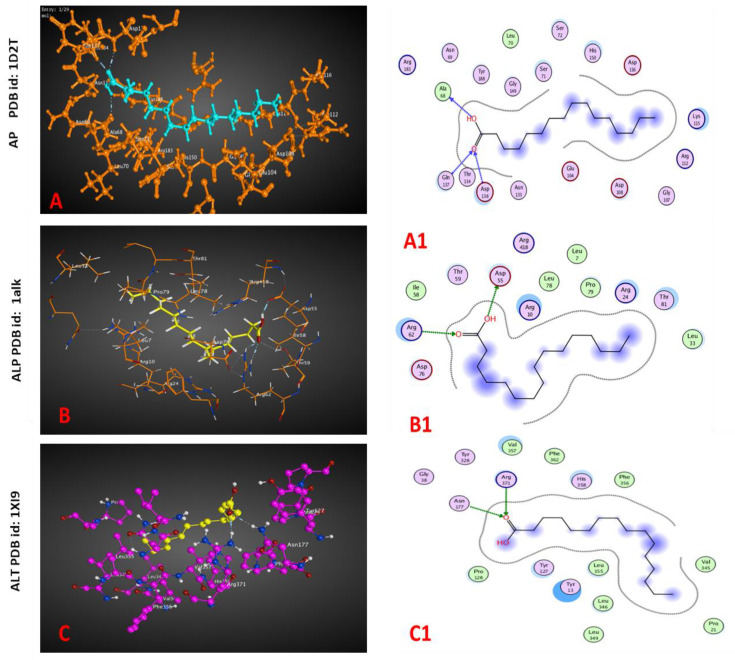
3D (**A**–**C**) and 2D (**A1**, **B1**, **C1**) docked interactions map for the palmitic acid **CH_3_** (**CH_2_**)**_14_COOH**) with the binding sites of acid (AP), alkaline phosphatases (ALP), and alanine aminotransferase (ALT).

The molecular docking revealed a potential interaction between the ligand molecule, palmitic acid, with acid, alkaline phosphatases, and ALT (Figure 3). There was an inhibitory action for the palmitic acid extracted from both plants against acid and alkaline phosphatases, while a reverse effect was detected with the ALT enzyme, as revealed by interaction-free energy, the docking score. In silico interaction ability was detected through H-acceptor scores (−4.3, −2.6, and −3.1 Kcal/mol) against acid and alkaline phosphatases and ALT enzymes, respectively (Table 9).

## 3. Discussion

*Calotropis procera* and *Atriplex halimus* are medicinal shrubs that can withstand harsh environmental conditions such as extreme heat, drought, and salinity [11,12]. They are also used in phytoremediation to remove heavy metals from soil [11,14]. Because of these factors, these plants are promising in terms of bioactive metabolite production as well as antimicrobial and anti-molluscicidal activity. The plants are from the genera *Calotropis* and *Atriplex*, both of which have demonstrated several biological activities.

The metabolic profiling of the methanolic extracts of *C. procera* and *A. halimus* was conducted using GC/MS. According to the present data, *C. procera* extract consists primarily of fatty acids and sterol metabolites, including palmitic acid, campesterol, stigmasterol, oleic acid, and stearic acid. Previous research reported by [17] found that the essential oil of the plant was predominantly composed of the metabolite’s phytol and linoleic acid. According to [18], the two most abundant fatty acids in the ethanolic leaf extract are palmitic acid and linoleic acid. A stigmasterol metabolite has been previously identified in the plant latex [19]. The biological activity and nutritional values of the metabolites identified in the plant extract have been previously characterized. Palmitic acid has been demonstrated to be cytotoxic to human leukemic cells while having no effect on healthy cells [20] and to possess antiviral activity [21,22]. Stigmasterol has been shown to reduce cholesterol levels and has additional health benefits, including protection against cancer, inflammation, and osteoarthritis [23]. It also proved to have larvicidal and antimicrobial properties [24,25]. Oleic acid has antioxidant properties, as reported by [26,27].

Herein, the extract of *C. procera* has a considerable concentration of the terpenoids phytol and spathulenol. The accumulation of terpenoid compounds in the plant oil extract has been previously reported [17]. Phytol and its derivatives were found to have anti-cancer, antioxidant, anti-pain, anti-inflammatory, immune-modulating, and antibacterial effects [28,29]. Spathulenol possesses an anti-inflammatory effect [30].

Our analysis of the metabolic profile of *A. halimus* confirmed the majority of methanolic extracts are fatty acids such as palmitic acid, oleic acid, and stearic acid, as well as sugar alcohols and sugars. Terpenoid metabolites made up a minor portion of the total. The qualitative study of the plant’s aerial part revealed the presence of flavonoids, polyphenols, and tannins [31]. Flavonol glycosides, phenolic glucosides, and methoxylated flavonoid glycosides were all isolated from the butanol extract from shoots [32]. The aqueous extract of the plant showed the presence of flavonoids [33]. LC/MS analysis of the plant ethanolic extract showed an abundance of phenolic compounds such as gallic acids and caffeic acid.

This study demonstrated that myo-inositol, D-pinitol, and xylonic acid are found in *A. halimus* extract and that these carbohydrates have biological and commercial value. Myo-inositol has been linked to numerous positive health effects in humans, including anti-diabetic and antioxidant properties, suppression of liver carcinogenesis, and alterations in mood state as a function of increased or decreased levels in the brain [34]. D-pinitol has been shown to protect the liver from lipid peroxidation, lower blood sugar, fight cancer, reduce inflammation, and function as an antioxidant [35]. Many different applications exist for xylonic acid, including chelation and use as a food additive. In addition to being a plasticizer and exhibiting high thermal stability, it also possesses a number of other useful properties [36].

In this study, five pathogenic bacterial species were tested for susceptibility to the antibacterial activity of methanolic extracts of *C. procera* and *A. halimus*. *C. procera* exhibited greater antibacterial activity than *A. halimus* in inhibiting the growth of *Escherichia coli*, *Pseudomonas aeruginosa*, and *Proteus mirabilis*. Moreover, *C. procera* was more effective than gentamycin against *Pseudomonas aeruginosa* and *Proteus mirabilis*. Both bacterial species are human pathogens: *Pseudomonas aeruginosa* is a major cause of illness in patients with cystic fibrosis, and it may cause persistent infections, largely due to its remarkable adaptability [37]; *Proteus mirabilis* is responsible for some urinary tract infections [38]. *Proteus mirabilis* has been shown to be resistant to various medicines, including colistin, in addition to showing decreased sensitivity to imipenem [39,40]. Studies on *C. procera* have shown that a methanol extract of the plant’s leaves has antibacterial properties against both Gram-positive and Gram-negative bacteria, including *Staphylococcus aureus, Bacillus subtilis, Escherichia coli,* and *Pseudomonas aeruginosa* [41]. The ethanolic extract of leaves showed antibacterial activity against *Escherichia coli* [18]. The development of *Salmonella typhi*, *Staphylococcus aureus,* and *Pseudomonas aeruginosa* has been shown to be inhibited by essential oils extracted from the plant’s leaves [18].

In the present study, both *C. procera* and *A. halimus* methanol extracts showed molluscicidal activity toward *B. alexandrina* snails, with the *C. procera* extract being more effective. The molluscicidal activities of *C. procera* extract against *Biomphalaria alexandrina* [42], *Biomphalaria arabica* snails [43], and *Schistosoma mansoni* [44] have been reported. Moreover, the plant oil showed antiparasitic activity against *Blastocystis* spp. [45]. Molluscicidal activities have been attributed to various members of the genus *Atriplex*. Methanolic extracts of *Atriplex glauca*, for instance, were found to significantly reduce the survival and growth rates of *B. alexandrina* snails. Meanwhile, *Atriplex inflata* demonstrated activity against *Galba truncatula* [46].

In the present investigation, the extracts from *C. procera* and *A. halimus* were toxic to adult *B. alexandrina* snails, lowered egg hatching success, and increased egg mortality. Additionally, these extracts caused high levels of DNA damage in the snails’ digestive glands. Snail DNA can be used as an indicator of toxicity, and the comet test is a highly accurate technique for identifying DNA damages, including single-strand breaks [47]. Both the oxidation of DNA nucleotides and the covalent bonding that forms with DNA are potential pathways for this genotoxicity; both of these mechanisms can contribute to strand breaks in DNA. Breaks in the DNA of aquatic species have been shown in previous studies to be connected with detrimental effects on a range of biological processes, including fertilization, the immunological response, development, and population dynamics [48].

Immunohistochemistry is a histological technique that determines the presence or absence of specific antigens in tissues by the use of specific immunological antibodies. Cyclin D1 is widely used as a histopathological marker in many tissues as it an important regulator of the cell cycle progression from G1 to S phase. In normal tissues, its expression is well regulated, while the highly expressed cyclin D1 might be a good surrogate to genotoxicity as it refers to the deregulation of the cell cycle [49,50]. In this research, adult snails that were exposed to sublethal concentrations of *C. procera* or *A. halimus* had anomalies in their digestive and hermaphrodite glands. These abnormalities included degenerations in digestive cells, ova, and sperm. Immunohistochemical analysis using cyclin D1 as a marker validated these changes. There was no expression of cyclin D1 in both glands of the control group, while 70% and 40% expression of cyclin D1 were observed in the interstitial cells after exposure to *C. procera* and *A. halimus* methanolic extracts, respectively. The molluscicidal activity of *C. procera* and *A. halimus* may be related to the presence of high levels of fatty acids, particularly palmitic acid, which has been reported to be responsible for the killing of *Pomacea canaliculata* snails [51]. The immunohistochemical analysis relies on specific interactions between antibodies and their target antigens [49,50]. Cyclin D1 controls how far along the cell cycle it is allowed to go. Increased cyclin D1 expression could enhance tumor growth by disrupting cell cycle control [52,53]. Bartkova et al. [54] proved that normal tissues have relatively low levels of cyclin D1 based on immunohistochemistry, and breast carcinomas exhibited overexpression and upregulation of cyclin D1.

Here, *B. alexandrina* snails were affected biochemically using sub-lethal doses (LC25) of methanol extracts of the plants that were studied. Both alkaline and acid phosphatase levels have gone down. The levels of total protein and albumin have also been lowered. The amount of alanine aminotransferase has been significantly increased. These findings are consistent with those of [55], in which they also recorded similar observations following chlorophyllin exposure to *B. alexandrina* snails. It has been reported that the toxicity of the snail species *Lanistes varicus* is correlated with decreased levels of protein, alkaline, and acid phosphatase [56]. It has been reported that the toxic effect of *Casimiroa edulis* and *Cestrum diurnum* plants on *B. alexandrina* is responsible for the modification in alanine aminotransferase activity [57].

Molecular docking is a potential method for investigating the activity of ligand compounds against the effects of some proteins via receptor–ligand interactions. Acid phosphatase is an enzyme incorporated in lysosomes and involved in autolysis and necrosis, while alkaline phosphatase has an important role in protein synthesis in gastropods [55]. The enzymatic mechanisms included acid phosphatase, alkaline phosphatase, and ALT. Our study showed that there were interactions between palmitic acid and acid, alkaline phosphatase, and ALT that might cause the inhibition of acid and alkaline phosphatase levels and lead to a decrease in the total protein concentration.

## 4. Materials and Methods

### 4.1. Plants

The healthy leaves of *Calotropis procera* and *Atriplex halimus* plants were collected from Wadi Degla Protectorate in Cairo, Egypt. *Atriplex halimus* was collected during the vegetative stage, while *Calotropis procera* was at the flowering stage in the summer of 2021 (June), the plants were collected early in the morning. Drought and extreme high temperatures characterize the summer season in Wadi Degla Protectorate. Three replicates were randomly obtained from three separate individuals for each species. The identification of the plants has been carried out according to [58].

A voucher plant specimen was kept at the herbarium of Helwan University’s Faculty of Science in Egypt.

#### 4.1.1. Metabolites Extraction

Fresh leaves of *Calotropis procera* and *Atriplex halimus* were allowed to air-dry in the shade. The leaves were ground into a fine powder. Briefly, five grams of powdered materials were extracted using 100 mL of 80% methanol at 50 °C for an hour while being stirred constantly. The samples were completely dried by evaporating the filtrate solutions at 40 °C until they were completely dry. The extract yield was estimated using the following formula: 100 (V/W), where V is the volume of dry extract and W is the weight of the plant material extracted. The yield of *Calotropis procera* was 0.70 g, while the yield of *Atriplex halimus* was 0.42 g.

#### 4.1.2. Gas Chromatography-Mass Spectroscopy (GC-MS) Analysis

**Derivatization and sample preparation**

Each dried polar residue from *C. procera* and *A. halimus* (three replicates from each plant) was combined with 80 µL of N, O-bis (trimethylsilyl) trifluoroacetamide silylation reagent (BFSTA) and 20 µL of trimethylchlorosilane (TMCS), and the mixture was then incubated for 1 h at 65 °C.

**GC/MS data collection and compounds identification**

The analysis of the metabolites was performed using a TRACE-GC ultra-gas chromatograph (Thermal Scientific Corp., Alvarado, TX, USA) coupled to a thermos mass spectrometer detector (ISQ single quadrupole mass spectrometer) and a 30 m × 0.32 mm i.d., 0.25 m film thickness, TR-5 MS column. With a split ratio of 1:10 and a flow rate of 1.0 mL/min, helium gas was used as the carrier gas. First, the temperature was adjusted to 60 °C for one min, and then it was gradually increased to 240 °C at a rate of 4.0 °C/min. Both the injector and the detector were maintained at a temperature of 210 °C. During the injection phase, 1 µL of the plant extract was diluted with hexane at a ratio of 1:10 hexane, *v*/*v*. Electron ionization (EI) at 70 eV was utilized to get mass spectra-spanning m/z ranges of 40–450. AMDIS, open-source software (www.amdis.net, accessed on 9 August 2022), Wiley’s spectrum library, and NSIT’s library databases were used to determine the identities of the metabolites.

### 4.2. The Antibacterial Activities

The antibacterial effect of plants methanol extracts was evaluated against one pathogen Gram-positive bacterium (*Staphylococcus aureus* ATCC 25923) and four Gram-negative pathogenic bacterial species, namely *Escherichia coli* ATCC 25922, *Pseudomonas aeruginosa* ATCC 7853, *Proteus mirabilis* ATCC 29906, and *Klebsiella pneumoniae* ATCC 700721. First, the nutrient agar (Diffco) medium was inoculated with the bacterial strains and placed in a 37 °C incubator. Each bacterium was then inoculated with a single colony and cultivated for 24 h at 37 °C in a nutrient broth medium. The antibacterial activities of plants’ extracts were studied using the well diffusion technique, as explained by [59]. Plates (9 mm) containing 20 mL of nutritional agar medium were inoculated with 100 µL of the bacterial suspension (1 × 106 CFU/mL). The agar plates were drilled into using a 6-mm cork borer to create wells. Each well had 100 µL of the plant extract at a concentration of 20 mg/mL. The plates were then incubated at 4 °C for 8 h, followed by 24 h at 37 °C. The well containing 100 µL of ethyl acetate was utilized as a negative control, while gentamycin (10 g/disc) was employed as a positive control in the experiment. Inhibition zones formed around the wells served as an indicator for the antibacterial efficacy. Inhibitory zone widths were then assessed in mm.

### 4.3. Molluscicidal Activity

#### 4.3.1. Snails

The methanolic extracts of *A. halimus* and *C. procera* leaves were tested for their molluscicidal properties against *B. alexandrina* snails. Snails’ average size was 8–10 mm, and they were acclimatized at the Laboratory of Medical Malacology at the Theodor Bilharz Research Institute (TBRI) in Giza, Egypt.

#### 4.3.2. Assessment of the Molluscicidal Activity of the Plant’s Methanol Extracts

Plants’ methanol extracts at different concentrations (95, 80, 65, 50, 35, and 25 mg/L) were prepared in order to determine the LC 10, 25, 50, and LC 90 at room temperature (22–25 °C) with a photoperiodicity of 12-h light/12-h dark [60]. Thirty replica snails of consistent size were utilized for each plant concentration, and another thirty identically sized snails were treated with dechlorinated water as a control. The snails were exposed to either plant extracts or dechlorinated water (for control) for 96 h. Then the snails were removed, rinsed with dechlorinated water, and given 24 h to recover. Dead snails were recorded as the average of the three replicates. Death of snails was distinguished by the immersion of snails in a small amount of 15–20% sodium hydroxide solution; if bubbles and blood come out of the snail, it is recorded as alive, and if not, it is recorded as dead [61].

#### 4.3.3. Effect of Plants Extract on Survival Rate of Snails

The snails, all of which measured between 8 and 10 mm in length, were randomly assigned to one of three groups: the first was exposed to the sub-lethal concentrations (LC_25_) of *C. procera* extract (127.8 mg/L), the second to the LC_25_ of *A. halimus* extract (204.5 mg/L), and the third to dechlorinated tap water as a control. Thirty snails were involved in each group. Subsequently, all samples were incubated in the test solution for a total of 24 h. Then, snails were collected, washed properly in dechlorinated water, and allowed to recover for 24 h in containers filled with fresh dechlorinated tap water. This process took two weeks. After recovery, snails were observed daily to record the survival rate for four weeks. The experiment was repeated thrice.

#### 4.3.4. Effect of Plants’ Extracts on Hatchability of Snails’ Eggs

Eggs were transferred to petri dishes, where they were exposed to the sub-lethal concentrations (LC_25_) of *Calitropis procera* (127.8 mg/L) and *Atriplex halimus* (204.5 mg/L). For each concentration, 100 eggs were used, and assays were repeated three times. At the end of the exposure period (24 h), eggs were transferred to petri dishes with dechlorinated water and examined daily under a stereomicroscope up to the 7th day.

### 4.4. Tissue Preparation

The soft tissues of the exposed and control groups were obtained by crushing the snail shells using two slides, weighing (1 g tissue/10 mL phosphate buffer), and homogenizing with a glass Dounce homogenizer. Then, the tissue homogenates were centrifuged (Sigma, 3–16PK, Germany) at 3000 rpm for 10 min, and the supernatants were stored at −80 °C until used.

#### 4.4.1. Biochemical Analysis

Bergmeyer’s approach [62], with some modifications by [63], was used to measure acid and alkaline phosphatases. Briefly, the tissue homogenate was quickly made by centrifuging it at 5000× *g* for 20 min at 4 °C after being immersed in ice-cold 0.9% NaCl (2% *w*/*v*). The levels of phosphatase activity were reported as mole/mg of tissue. This study used the protocol described in [64] to quantify total protein. Briefly, three tubes were prepared, and 5.0 mL of biuret reagent (cupric sulphate, sodium potassium tartrate, sodium hydroxide, and potassium iodide) was added to each tube. 100 µL of tissue homogenate solution (the sample) was added to the first tube. The second tube containing biuret reagent only was used as a blank. 100 µL of egg albumin was added to the third tube containing 5.0 mL of biuret reagent as a positive control (standard). Incubation for 30 min at 37 °C. To calculate the total protein concentration, the absorbance of the sample (A _Sample_) and standard (A _standard_) were measured against a reagent blank at 550 nm (520–570 nm). The following formula was used to calculate the protein concentration:

protein concentration (g/100 mL) = (A _Sample_/A _standard_) × 5

Analyses of albumin were conducted using the guidelines provided by [65]. Briefly, three tubes were prepared, and 2.0 mL of albumin reagent (citrate buffer, pH 4.2, bromcresol green, detergent, and preservative) was added to each tube. An amount of 10 µL of tissue homogenate solution (the sample) was added to the first tube. The second tube containing albumin reagent only was used as a blank. As a positive control (standard), 10 µL of albumin was added to the third tube containing 2.0 mL of albumin reagent. Incubation for 5 min. at 37 °C. To calculate the albumin concentration, the absorbance of the sample (A _Sample_) and standard (A _standard_) against reagent blank at 630 nm were measured. The calculation formula was as follows:

Albumin concentration (g/100 mL) = (A _Sample_/A _standard_) × 4.

The alanine aminotransferase levels were measured using the [66] technique. Briefly, 1 mL of the tissue homogenate is pipetted into a test tube and incubated in a water bath at a constant temperature of 40 °C for 10 min. An amount of 200 µL of serum was added and mixed well, and after an incubation period of exactly 30 min, the tube was removed from the water bath. A total of 1 mL of 2, 4-dinitrophenylhydrazine reagent was added to allow the reaction to be terminated. The tube was permitted to stand at room temperature for a minimum of 20 min, then 10 mL of 0.4 N sodium hydroxide was added, and the contents were well mixed. This mixture was left for exactly 30 min, and the optical density of the solution was measured at 505 nm using water as the blank. The number of units/liters was determined by using a standard curve.

#### 4.4.2. Comet Assay

The *B. alexandrina* snails were subjected to *A. halimus* or *C. procera* methanolic extracts at LC_25_ of 204.5 mg/L or 127.8 mg/L, respectively, for 24 h, then the snails were dissected and their head-foot regions were frozen at −80 °C. DNA damage was quantified using a single-cell gel technique, as published by [67]. Briefly, the tissues of control and exposed snails were cut into small pieces in phosphate buffer saline, then centrifuged 500× *g* for 5 min. The resulting supernatant was kept, and the pellets were discarded. The supernatant then centrifuged with a high speed 10,000× *g* to concentrate the cells, keep the pellets, and discard supernatant. Add 20 µL of the pellets to 180 µL of low melting agarose (0.5%). Take a drop of this mixture and put it on a slide then cover it and place it on ice. Leave for 15 min to ensure the freezing of the gel, then remove the coverslip and the slides and put them in a sectioned box that contains a lysis buffer (each 1 L contains 2.5 M NaCl, 100 mM EDTA, 8 g of NaOH, and 10 mM trisabase). Leave it in the fridge for 24 h. For DNA damage visualization, observations are made of EtBr-stained DNA using a 40× objective on a fluorescent microscope. Coding and scoring the slides were performed separately.

#### 4.4.3. Histopathological and Immunohistochemical Analysis

Adult *B. alexandrina* snails were subjected to LC_25_ (204.5 mg/L) or LC_25_ (127.8 mg/L) of either *A. halimus* or *C. procera,* respectively, for 24 h, followed by a two-week recovery period. According to the findings given by [68], the digestive and hermaphroditic glands were collected and processed. After cutting the tissues and immersing them in 10% formalin for 12 h, they were then dehydrated in ethanol at ascending concentrations of 80%, 90%, and 100% for 3 h each, cleaned in xylene, and then embedded in paraffin. After being cut on a microtome into 5-micrometer-long segments, the samples were mounted on slides, then dewaxed in xylene, stained with haemoxylin and eosin, and finally coated with Canada balsam. Utilization of a Zeiss microscope for the purpose of conducting an analysis on stained slides (Carl Zeiss Microscopy GmbH, 07, 745 Jena, Germany). Prior to undergoing immunohistochemistry analysis, adult snail tissue was sliced to a thickness of 4 mm and then mounted on slides that had been given a positive charge (Super Frost Plus, Menzel-Glaser, Germany). The slides were stained using anti-mouse proliferating cell antigen (PCNA) and cyclin D1 antibodies that were bought from Santa Cruz Biotechnology in the United States of America. This process took place on an automated platform. These antibodies performed most effectively when diluted at a ratio of 1:100. In order to calculate the percentages of positively stained brown nuclear material (PCNA, Cyclin D1), calculations were determined under Zeiss light microscopy at 400× magnification power.

### 4.5. The Molecular Docking Study

To explore the effect of exposing snails to *C. procera* and *A. halimus* methanolic extracts, acid and alkaline phosphatases and ALT enzymes were selected to predict their action with palmitic acid, a compound found in the GC analysis of both plant extracts. Using the Protein Data Bank (PDB), the molecular structure of tested enzymes was obtained and encoded, including acid phosphatase (1D2T) from *Escherichia blattae* [69], alkaline phosphatases (1alk) from *Escherichia coli* [70], and ALT (1XI9) from *Pyrococcus furiosus* [71]. Molecular docking was conducted using Molecular Operating Environment software (MOE 2014.09). The energy of the ligand palmitic acid compound was minimized, and after choosing the correct sequence of enzymes, hydrogens were added, and partial charges were calculated.

### 4.6. Statistical Analysis

All the experiments were randomly designed At least three replicates from each treatment were used, and Probit analysis was conducted to calculate the lethal concentration of *A. halimus* or *C. procera* extracts against *Schistosoma mansoni*. Minitab 17 was utilized to conduct the one-way ANOVA analysis of the data. Means between treatments were compared using the 95% confidence interval of the Fisher least significant difference (LSD) method.

## 5. Conclusions

This study demonstrated that methanolic extracts of the medicinal shrubs *Calotropis procera* and *Atriplex halimus* are rich in fatty acids (both saturated and unsaturated), glucosides, and sterols. *Calotropis procera* extract was more effective against *Escherichia coli* ATCC 25923, *Pseudomonas aeruginosa* ATCC 7853, and *Proteus mirabilis* ATCC 29906 than *Atriplex halimus*. Both plant extracts were found to have molluscicidal activity against *Biomphalaria alexandrina* snails, as determined by a variety of tests, including the mortality rate of adult snails, the hatchability rate of eggs, biochemical and histological analyses, and visual examinations of snail tissue. Based on the data, it was clear that *Calotropis procera* extract had greater anti-molluscicidal activity.

Our next studies will be to investigate the effect of pure compounds isolated from the plants under study in order to identify the metabolites responsible for the molluscicidal activity as an intriguing approach to eliminating schistosomiasis. Furthermore, because of the broad-spectrum effects, the safety of the tested metabolites will be assessed against other (non-target) organisms such as the water flea, *Daphnia magna*, which is extremely sensitive to water pollution. Measurements of many digestive and hermaphrodite gland enzymes will be used to investigate the sub-lethal effects on snail fecundity/fertility, and cercariae.

## Figures and Tables

**Table 1:** List of metabolites identified in the methanolic extract of *Calotropis procera*.

	Compound Name	Molecular Formula	Molecular Weight (g/mol)	Retention Time (min)	Area %
1	Formaldehyde	CH_2_O	30.02	4.16	1.3
2	Pentitol	C_5_H_12_O_5_	152.15	5.42	0.3
3	L-Valine	C_5_H_11_NO_2_	117.15	6.59	0.64
4	L-Leucine	C_6_H_13_NO_2_	131.17	8.25	0.28
5	L-Isoleucine	C_6_H_13_NO_2_	131.17	8.76	0.35
6	Glycerol	C_3_H_8_O_3_	92.09	11.37	2.65
7	Butanedioic acid	C_4_H_6_O_4_	118.09	12.35	6.19
8	Malic acid	C_4_H_6_O_5_	134.09	16.76	2.34
9	L-Proline	C_5_H_9_NO_2_	115.13	17.33	1.21
10	DL-Phenylalanine	C_9_H_11_NO_2_	165.19	17.89	0.83
11	Spathulenol	C_15_H_24_O	220	18.71	0.71
12	Methyl-β-D-glucopyranoside	C_7_H_14_O_6_	194.18	20.48	0.26
13	L-Fucitol	C_6_H_14_O_5_	166.17	22.42	0.58
14	4-Coumaric acid	C_9_H_8_O_3_	164.16	23.36	0.23
15	Azelaic acid	C_9_H_16_O_4_	188.22	23.61	0.21
16	D-Fructofuranose	C_6_H_12_O_6_	180.16	23.80	2.40
17	D-Tagatofuranose	C_6_H_12_O_6_	180.16	23.97	0.45
18	Methyl D-glucofuranoside	C_7_H_14_O_6_	194.18	24.60	0.75
19	α-L-Arabinopyranose	C_5_H_10_O_5_	150.130	24.68	0.32
20	Dulcitol	C_6_H_14_O_6_	182.17	25.51	0.36
21	α-d-glucopyranose	C_6_H_12_O_6_	180.16	25.86	0.52
22	Hexadecanoic acid, methyl ester	C_17_H_34_O_2_	270.4	26.19	0.23
23	D-Xylofuranose	C_5_H_10_O_5_	150.13	26.96	0.54
24	α-D-Allopyranose	C_6_H_12_O_6_	180.16	27.41	0.31
25	D-Allofuranose	C_6_H_12_O_6_	180.16	27.60	0.45
26	Palmitic Acid	C_16_H_32_O_2_	256.42	28.52	10.74
27	10-Octadecenoic acid, methyl ester	C_19_H_36_O_3_	312.5	29.47	0.51
28	Heptadecanoic acid	C_17_H_34_O_2_	270.5	30.31	0.32
29	Phytol	C_20_H_40_O	296.5	30.82	3.91
30	9,12-Octadecadienoic acid (alpha-Linoleic acid)	C_18_H_32_O_2_	280.4	31.42	1.79
31	Oleic Acid	C_18_H_34_O_2_	282.46	31.54	8.04
32	Petroselinic acid	C_18_H_34_O_2_	282.46	31.68	0.92
33	Stearic acid	C_18_H_36_O_2_	284.48	32.04	4.24
34	5,8,11-Eicosatriynoicacid	C_20_H_28_O_2_	300.4	32.76	0.30
35	D-Trehalose	C_12_H_22_O_11_	342.3	36.63	3.23
36	D-(+)-Turanose	C_12_H_22_O_11_	342.3	37.15	1.82
37	Sucrose	C_12_H_22_O_11_	342.3	37.61	3.29
38	Dasycarpidan-1-methanol, acetate (ester)	C_20_H_26_N_2_O_2_	326.4	39.17	1.46
39	Trilinolein	C_57_H_98_O_6_	879.4	39.81	0.25
40	Dasycarpidan-1-methanol, acetate (ester)	C_20_H_26_N_2_O_2_	326	40.02	0.52
41	Oleic acid, eicosyl ester	C_38_H_74_O_2_	562.9	40.47	0.41
42	Ser-Asp-Gly-Arg-Gly	C_17_H_30_N_8_O_9_	490	41.47	0.96
43	Ursolic aldehyde	C_30_H_48_O_2_	440.7	42.45	1.31
44	2-Butenoic acid, 2-methyl-, 2-(acetyloxy)-1,1a,2,3,4,6,7,10,11,11 a-decahydro-7,10-dihydroxy-1,1,3,6, 9-pentamethyl-4a,7a-epoxy-5H-cyclo penta[a]cyclopropa[f]cycloundecen-1 1-yl ester	C_27_H_38_O_8_	490	43.02	0.22
45	α-Tocopherol	C_29_H_50_O_2_	430.7	43.11	1.34
46	L-Arabinitol pentaacetate	C_15_H_22_O_10_	362	43.44	0.29
47	α-Carotene	C_40_H_56_	536	43.98	1.76
48	Campesterol	C_28_H_48_O	400.7	44.67	8.13
49	Stigmasterol	C_29_H_48_O	412.7	45.08	8.48
50	Oleyl oleate	C_36_H_68_O_2_	532.9	45.41	1.41
51	(Z)-Icos-11-en-1-yl oleate	C_38_H_72_O_2_	560.9	45.59	0.43
52	2-Hydroxy-3-[(9E)-9-octadecenoyloxy] propyl	C_39_H_72_O_5_	620.5	45.76	0.22
Total					90.7%

**Table 2:** List of metabolites identified in the methanolic extract of *Atriplex halimus*.

	Compound Name	Molecular Formula	Molecular Weight	Retention Time (min)	Area %
1	Propionic acid	C_3_H_6_O_2_	74.07	5.98	0.65
2	Glycolic acid	C_2_H_4_O_3_	76.05	6.35	0.35
3	L-Alanine	C_3_H_7_NO_2_	89.09	6.97	1.18
4	Hydracrylic acid	C_3_H_6_O_3_	90.08	7.99	0.26
5	L-Valine	C_5_H_11_NO_2_	117.15	9.77	0.31
6	Urea	CH_4_N_2_O	60.05	10.58	0.85
7	Glycerol	C_3_H_8_O_3_	92.09	11.37	3.43
8	L-Proline	C_5_H_9_NO_2_	115.13	11.79	0.64
9	Butanedioic acid	C_4_H_6_O_4_	118.09	12.35	2.04
10	L-Serine	C_3_H_7_NO_3_	105.09	13.56	0.38
11	Homoserine	C_4_H_9_NO_3_	119.12	15.78	0.23
12	L-5-Oxoproline	C_5_H_7_NO_3_	129.11	17.34	1.58
13	L-Aspartic acid	C_4_H_7_NO_4_	133.1	17.49	0.23
14	Methyl alpha-D-galactopyranoside	C_7_H_14_O_6_	194.18	17.75	0.32
15	L-Threonic acid	C_4_H_8_O_5_	136.1	18.06	0.26
16	2,3,4-Trihydroxybutyric acid	C_4_H_8_O_5_	136.1	18.49	0.76
17	Spathulenol	C_15_H_24_O	220.35	18.71	0.61
18	L-Asparagine	C_4_H_8_N_2_O_3_	132.12	19.20	0.45
19	Pentanedioic acid	C_5_H_8_O_4_	132.11	19.37	0.36
20	L-Phenylalanine	C_9_H_11_NO_2_	165.19	19.76	0.21
21	Xylonic acid	C_5_H_10_O_6_	166.13	19.86	0.91
22	D-(+)-Arabitol	C_5_H_12_O_5_	152.15	21.51	0.36
23	L-Fucitol	C_6_H_14_O_5_	166.17	22.41	0.28
24	Ribonic acid	C_5_H_10_O_6_	166.13	22.68	0.75
25	L-(+)-Tartaric acid	C_4_H_6_O_6_	150.09	22.93	0.28
26	D-Xylofuranose	C_5_H_10_O_5_	150.13	23.06	0.30
27	D-Pinitol	C_7_H_14_O_6_	194.18	23.14	1.63
28	α -D-Glucopyranosiduronic acid	C_42_H_71_NO_19_	894	23.61	0.60
29	D-(-)-Fructofuranose	C_6_H_12_O_6_	180.16	23.80	2.72
30	D-Psicofuranose	C_6_H_12_O_6_	180.16	23.9	2.54
31	Citric acid	C_6_H_8_O7	192.12	24.10	4.05
32	Myo-Inositol	C_6_H_12_O_6_	180.16	24.25	5.14
33	Methyl-D-glucofuranoside	C_7_H_14_O_6_	194.18	24.60	2.20
34	D-Mannonic acid	C_6_H_12_O_7_	196.16	24.98	1.52
35	α-D-(+)-Talopyranose	C_6_H_12_O_6_	180.16	25.54	1.51
36	1,5-Anhydrohexitol	C_6_H_12_O_5_	164.16	25.69	0.32
37	α –Lyxopyranose	C_5_H_10_O_5_	150.13	25.86	0.61
38	Methyl palmitate	C_17_H_34_O_2_	270.5	26.20	0.75
39	D-Lyxofuranose	C_5_H_10_O_5_	150.13	26.95	1.03
40	D-(+)-Talofuranose	C_6_H_12_O_6_	180.16	27.75	0.44
41	Palmitic Acid	C_16_H_32_O_2_	256.42	28.52	6.47
42	D-Allofuranose	C_6_H_12_O_6_	180.16	28.92	0.42
43	Linoleic acid ethyl ester	C_20_H_36_O_2_	308	29.32	0.80
44	*cis*-13-Octadecenoic acid, methyl ester	C_19_H_36_O_2_	296	29.46	1.21
45	Methyl stearate	C_19_H_38_O_2_	298	29.98	0.36
46	Phytol	C_20_H_40_O	296.5	30.82	0.25
47	9,12-Octadecadienoic acid	C_18_H_32_O_2_	280.4	31.42	1.55
48	Oleic Acid	C_18_H_34_O_2_	282.5	31.54	5.25
49	cis-11-Octadecenoic acid	C_18_H_34_O_2_	282.5	31.68	0.82
50	Stearic acid	C_18_H_36_O_2_	284.5	32.05	4.01
51	Linoelaidic acid	C_18_H_32_O_2_	280.4	33.07	0.32
52	D-(+)-Galacturonic acid	C_6_H_10_O_7_	194.14	34.36	0.33
53	11-Eicosenoic acid	C_20_H_38_O_2_	310.5	34.86	0.42
54	á-D-Galactopyranoside	C_6_H_12_O_6_	180.16	35.30	0.33
55	Sucrose	C_12_H_22_O_11_	342.3	36.63	2.24
56	D-Trehalose	C_12_H_22_O_11_	342.3	37.15	0.85
57	Oleic acid, eicosyl ester	C_38_H_74_O_2_	562	39.10	0.45
58	Dasycarpidan-1-methanol, acetate (ester)	C_20_H_26_N_2_O_2_	326	39.17	0.19
59	Fumaric acid	C_4_H_4_O_4_	116.07	39.22	0.19
60	2-Oleoylglycerol	C_21_H_40_O_4_	356.5	39.32	0.42
61	2-Hydroxy-3-[(9E)-9-octadecenoyloxy]propyl(9E)-9-octadecenoate	C_39_H_72_O_5_	620	40.03	0.45
62	Dasycarpidan-1-methanol, acetate (ester)	C_20_H_26_N_2_O_2_	326	40.74	0.54
63	9-Octadecenoic acid, (2-phenyl-1,3-dioxolan-4-YL) Methyl ester	C_28_H_44_O_4_	444	43.14	0.50
64	Stigmasterol	C_29_H_48_O	412.7	45.06	0.36
65	(Z)-Icos-11-en-1-yl oleate	C_38_H_72_O_2_	560	45.39	1.11
66	E,E,Z-1,3,12-Nonadecatriene-5,14-d Iol	C_19_H_34_O_2_	294	45.62	0.83
	Total				73.7%

**Table 3:** The antibacterial activities of *Atriplex halimus* and *Calitropis procera* leaves extract.

Bacterial Species	Inhibition Clear Zone Diameter (mm)			
	*Atriplex halimus*	*Calitropis procera*	Gentamycin (10 μg/disc)	Ethyl Acetate
*Staphylococcus aureus* ATCC 25923	-ve	-ve	17 ± 0.2	-ve
*Escherichia coli* ATCC 25922	-ve	10 ± 0.1 ^b^	15 ± 0.8 ^a^	-ve
*Pseudomonas aeruginosa* ATCC 7853	14 ± 0.5 ^c^	18 ± 0.3 ^a^	17 ± 0.6 ^b^	-ve
*Proteus mirabilis* ATCC 29906	-ve	18 ± 0.2 ^a^	10 ± 0.0 ^b^	-ve
*Klebsiella pneumoniae* ATCC 700721	-ve	-ve	12 ± 0.5	-ve

The letters (a, b, c) assigned to each column indicates the significance between mean of the group being compared at *p* < 0.05 level according to Fisher test. Therefore, columns followed by different letters (a, b, c), indicate that the mean values in these columns are significantly different from each other.

**Table 4:** Molluscicidal activity of the methanolic extracts of *Atriplex halimus* and *Calitropis procera* leaves against *B. alexandrina* snails.

Slope	LC_90_ (mg/L)	LC_50_ (mg/L)	LC_25_ (mg/L)	LC_10_ (mg/L)	Plants
1.1	260.4	223.8	204.5	187.2	*Atriplex halimus*
1.0	148.5	135	127.8	121.4	*Calitropis procera*

**Table 5:** Survival rate of *B. alexandrina* snails exposed to sub lethal concentrations LC_25_ of *C. procera* (127.8 mg/L) and *A. halimus* (204.5 mg/L) methanolic extracts.

Weeks	Survival Rate (%)		
	Control	*A. halimus*	*C. procera*
1	99 ^c^	80 ^b^	55 ^a^
2	95 ^c^	60 ^b^	30 ^a^
3	95 ^c^	40 ^b^	15 ^a^
4	90 ^c^	20 ^b^	5 ^a^

The letters (a, b, c) assigned to each column indicates the significance between mean of the group being compared at *p* < 0.05 level according to Fisher test. Therefore, columns followed by different letters (a, b, c), indicate that the mean values in these columns are significantly different from each other.

**Table 6:** Hatchability and mortality rates of *Biomphalaria alexandrina* snail’s eggs exposed to sub lethal concentrations LC_25_ of *Calitropis procera* (127.8 mg/L) and *Atriplex halimus* (204.5 mg/L) methanolic extracts for 24 h.

Group	% Hatchability	% Mortality
Control	100 ^c^	0 ^c^
*Atriplex halimus*	60 ^b^	40 ^b^
*Calitropis procera*	30 ^a^	70 ^a^

The letters (a, b, c) assigned to each column indicates the significance between mean of the group being compared at *p* < 0.05 level according to Fisher test. Therefore, columns followed by different letters (a, b, c), indicate that the mean values in these columns are significantly different from each other.

**Table 7:** DNA single strand breaks after exposure of *B. alexandrina* snails to sub-lethal concentrations of *A. halimus* and *C. procera* methanolic extractions.

	Olive Tail Moment	Tail Length (px)	% DNA in Tail	Tail Moment
Control	1.71	4.62 ± 0.58 ^c^	16.39 ± 4.25 ^b^	0.94 ± 0.31 ^c^
*Atriplex halimus* (LC_25_)	2.11	6.24 ± 0.12 ^b^	16.21 ± 1.11 ^b^	1.23 ± 1.13 ^b^
*Calotropis procera*(LC_25_)	2.99	8.35 ± 0.92 ^a^	20.25 ± 0.21 ^a^	2.14 ± 0.72 ^a^

The letters (a, b, c) assigned to each column indicates the significance between mean of the group being compared at *p* < 0.05 level according to Fisher test. Therefore, columns followed by different letters (a, b, c), indicate that the mean values in these columns are significantly different from each other.

**Table 8:** The biochemical effects on *B. alexandrina* snails exposed to sublethal concentrations LC_25_ of *C. procera* (127.8 mg/L) and *A. halimus* (204.5 mg/L) methanolic extracts.

	Alkaline Phosphatase (μmole/mg)	Acid Phosphatase (μmole/mg)	Total Protein g/100 mL	Albumin g/100 mL	Alanine Aminotransfersa (ALT) U/L
Control	105.7 ± 0.05 ^c^	125 ± 0.2 ^b^	5.8 ± 0.11 ^b^	3.4 ± 0.1 ^b^	68.2 ± 0.5 ^c^
LC_25_ *Atriplex halimus*	75.4 ± 0.1 ^b^	95.2 ± 0.4 ^a^	3.9 ± 0.12 ^a^	3.1 ± 0.1 ^b^	88.5 ± 0.6 ^b^
LC_25_ *Calotropis procera*	60.5 ± 0.3 ^a^	80.5 ± 0.2 ^a^	3.6 ± 0.23 ^a^	2.4 ± 0.3 ^a^	107.2 ± 0.4 ^a^

The letters (a, b, c) assigned to each column indicates the significance between mean of the group being compared at *p* < 0.05 level according to Fisher test. Therefore, columns followed by different letters (a, b, c), indicate that the mean values in these columns are significantly different from each other.

**Table 9:** In silico docking study of acid, alkaline phosphatases, and the hepatopancreas enzyme, ALT with palmitic acid as a ligand.

PDB ID	Docking Score (Kcal/mol)	Interaction Type	Amino Acid Residue Involved in Docking
AP (1D2T)	−1.1	H-donor H-acceptor H-acceptor	ALA 68
	−4.3		GLN 137
	−2.0		ASP 138
ALP (1alk)	−6.3	H- donor H-acceptor H-acceptor	ASP 55 ARG 62 ARG 62
	−2.6		
	−2.1		
ALT (1XI9)	−0.7 −3.1	H-acceptor H-acceptor	ASN 177 ARG 371

## Data Availability

All data generated or analyzed during this study are included in this article.
